# Towards patient-relevant structures: reviewing body-temperature biological macromolecules and their ligands for pharmaceutical applications

**DOI:** 10.1107/S2059798325010617

**Published:** 2026-01-01

**Authors:** Alice Brink, John R. Helliwell, Francois J. F. Jacobs

**Affiliations:** a Chemistry Department University of the Free State Nelson Mandela Drive Bloemfontein South Africa; b Department of Chemistry University of Manchester Oxford Road Manchester United Kingdom; Osaka University, Japan

**Keywords:** body temperature, physiological conditions, 37°C, diffraction structural biology, PDB–CSD survey, thermophiles, hyperthermophiles

## Abstract

The aim of our structures for eventual clinical application is to be relevant. Regulation of pharmaceutical lead compounds, however, does not yet involve the need for patient-relevant macromolecular structures determined at 37°C, as it is not yet known whether crystal growth and diffraction at 37°C versus standard cryo-condition practices will reveal significant binding variations applicable for drug development or, in the case of extremophiles, provide insight into their function. However, for select examples in the literature interesting changes occurred, and support the initiative that data collection at high temperatures should be considered. This topical review considers a Protein Data Bank (PDB) and Cambridge Structural Database (CSD) data survey of crystal structures that have been determined at elevated temperatures, *i.e.* neither under cryogenic conditions nor at typical room-temperature conditions of 20–25°C, and reveals a few hurdles as well as many successes in reaching such patient-relevant structures. It highlights key methodology that appears in the literature which could benefit those considering related research. Since it is possible for crystallographic structure-determination methods to be adapted to 37°C, amid some challenges, we encourage the initiative that many more could be determined at 37°C. Included in the studies deposited in the PDB are some that have been performed at temperatures in excess of >37°C, and surprisingly several at even higher temperatures (*i.e.* 50–90°C). The overall aim of determining the 3D structure of a biological macromolecule at its natural body temperature has in principle to include crystallization and diffraction data collection. In the survey we find very few crystallizations performed at 37°C followed by data collection at the same temperature, and few have conducted a systematic study of comparing the changes occurring at 100 K versus 37°C. It is of course assumed that some key drug binding in proteins may occur over a narrow temperature range appropriate for mesophilic organisms, whereas for thermophilic organisms the protein may well exist over a wide temperature range reflecting that in which the organism is able to thrive. For the higher temperature structure solutions, those in the range which is more appropriate for thermophiles or hyperthermophiles, no crystallizations at these extreme temperatures have yet been conducted. The ability to conduct crystallization at 37°C and obtain acceptable high-resolution data at the same temperature is surely encouraging to the crystallographic community to build on these achievements for this and the full temperature range. We describe aspects of crystallization, mounting and transfer of crystals, data collection, reporting of metadata within databases *etc.* that have been notable during the survey of the data and highlight them here for the benefit of the community which may be considering 37°C data analysis from pre-crystal growth to re-refinement of data. For comparable data and to avoid any experimental bias, we also encourage the community to complete the analysis sequentially, as few have considered this holistic analysis of solid-state variations which may occur over the low-to-high temperature range.

## Abstract 2

Regulation of pharmaceutical lead compounds does not yet involve the need for patient-relevant macromolecular structures determined at 37°C; nevertheless, we highlight the advantages of these to ensure chemical relevance and the identification of changes which may occur under physiological conditions. The PDB and CSD entries for diffraction data measured at ≥37°C, as well as a cryoEM 37°C freeze-trapped structure, are considered and the description includes structures obtained well above 37°C that are relevant to thermophile and hyperthermophile structural biology.

## 1. Introduction

There is a growing trend of measuring diffraction data at 37°C and above to identify atomic movement, which may become prominent at higher temperatures. Our specific motivation is to seek physiologically relevant structures of rhenium compounds bound to a protein, where the rhenium is a chemical model for technetium applicable for theranostic (therapy or diagnostics) clinical purposes (Jacobs *et al.*, 2024 ▸), with the specific understanding that the kinetic reactivity rate constants for organometallics can double for each 10°C increase in temperature. A structural perspective on the temperature-dependent activity of enzymes has recently been described by McLeod *et al.* (2025 ▸). We have also reviewed the activity of macromolecular crystallography in the domain of ≥37°C structures with respect to other biophysics methods (Brink *et al.*, 2025 ▸) and find that the methodology within macromolecular crystallography is perhaps less ideally suited to high temperature than these other methods, especially NMR, which has extensively been used to study protein unfolding in solution (Fersht, 1999 ▸). Naturally, NMR has the advantage of analysis being conducted in the solution state and is less dependent on the formation of a single crystal or radiation damage which takes place during X-ray diffraction analysis, a guiding consideration for cryo-data collection. There is macromolecular crystallography research aimed at understanding enzyme structure and mechanism in which a combination of time-resolved sequences are combined with a variation of temperature above 37°C as well as below (see, for example, Schulz *et al.*, 2025 ▸). There are also a range of individual motivations such as in nanoscience (Bailey & Tezcan, 2020 ▸) and chemical biology (Paolillo *et al.*, 2025 ▸; Tito, Ferraro, Garribba *et al.*, 2025 ▸; Tito, Ferraro & Merlino, 2025 ▸). Recent reviews of complementary biophysics methods as well as X-ray crystallography have been published for this higher temperature range (Brink *et al.*, 2025 ▸; Ferraro & Merlino, 2025 ▸). There are also excellent descriptions of how to practically grow crystals and conduct these experiments (Fischer, 2021 ▸; Hough *et al.*, 2023 ▸; Thorne, 2023 ▸), to which the reader is referred. There is a classic study (Jollès & Berthou, 1972 ▸) of the high-temperature crystallization of lysozyme, and a series of follow-up papers involving the crystallization of hen egg-white lysozyme at 310 K and with an X-ray crystal structure study, PDB entry 1bgi (Oki *et al.*, 1999 ▸). The overall motivation of Jollès & Berthou (1972 ▸) is described in their associated paper (Jollès *et al.*, 1975 ▸), where they observe that ‘it is well known that the bacterial substrate of lysozyme is lysed more rapidly at 37°C or 40°C than at 20°C’ and that ‘many kinetical studies devoted to enzymes should be reexamined at more physiological conditions’. Extending the vision of Jolles and Berthou to structural 3D studies at 37°C thus seems quite natural.

This article reviews crystal structures that have been determined at elevated temperatures, *i.e.* neither under cryogenic conditions nor at typical room-temperature conditions of 20–25°C. From our survey of current PDB structures where the crystals were grown or analysed at 37°C we highlight a few key observations which occurred during the survey of the data and which are additionally relevant. Naturally, there are safeguards to support current 3D cryo structure results and which confirm ligand–protein binding, such as binding assays performed at 37°C. Such assays can involve one or all of inhibition of reaction assays, mass-spectrometric measurements and IR or NMR assessment of weak binders. New developments include the OpenBind consortium (https://openbind.uk/), which will utilize machine-learning tools to combine automated crystallography and microscale chemistry to accelerate drug discovery by generating protein–ligand structure and affinity data sets.

## 2. PDB data surveys

The crystallographic databases (PDB and CSD) have achieved a remarkable surety and range of metadata for each deposition. This allows the ready assembly of core data relevant to a topical review such as this. In Supplementary Tables S1, S2 and S3 we survey the PDB for all depositions with X-ray diffraction data measured at or above 37°C, with 51 PDB data collections. Supplementary Table S1 contains entries at 37°C alone, or temperatures close to it (*i.e.* 40°C). Supplementary Table S2 contains macromolecular crystal structures deposited in the PDB for data measured well above 37°C. Supplementary Table S3 is for a macromolecular structure determined by cryoEM where the sample was flash-frozen at 37°C. It is kept separate from Supplementary Table S1 because the metadata are necessarily different.

For the macromolecular model refinement of the listed PDB entries, a variety of calculational methods have been employed. Mainly, these treat the structure as a conventional model refinement, but there is evident enthusiasm for ensemble model refinement, which to the best of our knowledge began in earnest during the early 2000s (van den Bedem *et al.*, 2009 ▸). The crystallographic data in this review were re-examined with *PDB-REDO* (Joosten *et al.*, 2014 ▸) to ensure a baseline of uniformity for calculation methods despite the age of the deposited data or the software originally used. This is a very useful procedure to evaluate structures re-refined with various software using the most up-to-date methods. The *PDB-REDO* maps and models have been scrutinized explicitly and while refinement showed a few modelling corrections, it was noted that a number of bound waters placed by the depositors have been stringently removed from the depositors’ models, as shown by the *PDB-REDO* (*F*_o_ − *F*_c_) difference Fourier electron-density map peaks above 5σ list, which however clearly indicates that a good fraction of these could be reinstated according to this criterion. Discussion with the PDB-REDO team (Robbie Joosten, personal communication) indicated that they are planning to review their software dealing with assessing and pruning depositors’ bound waters. We recall the importance of waters being modelled for drug development (Zsidó & Hetényi, 2025 ▸) and the substitution of ligands often occurring at former water sites, such as those described in lectin–carbohydrate complex structures (Modenutti *et al.*, 2015 ▸; Levitt & Park, 1993 ▸). When these latter are displaced they thereby make a favourable entropic contribution to the Gibbs free-energy change, favouring the binding of a ligand. The value of machine learning for classifying water/ions sites could greatly assist in identifying potential sites (Shub *et al.*, 2025 ▸).

A survey of available PDB entries re-examined with current software can run the risk of artificial deletion. The main study at risk from the current deletion of bound waters is that of Chevrier *et al.* (1986 ▸), whose main finding was stated as follows:

The main difference resides in a more extensive hydration shell in the crystal grown at high temperature than in the crystal grown at low temperature.

*PDB-REDO* made each model from Chevrier *et al.* (1986 ▸) have a very similar number of bound waters (35 versus 39), and viewing the structures in *Coot* (Emsley *et al.*, 2010 ▸) suggests that this main finding of the effect of more waters at 310 K is possibly artificially removed in such a case by the re-refinement.

Additionally, it was noted that studies of macromolecular structures at 37°C and above may well need more ensemble modelling and reviewers should be considerate of the higher thermal displacement of atoms in the overall increasing dynamics of the crystal structure, which will not be commonly observed in standard 100 K data. This aspect was noted in the freeze-trap cryoEM study, where the sample was initially held at 37°C (Hu *et al.*, 2024 ▸), and illustrates the temperature-dependent changes in the structure and function of TRPM4; there is an excellent video illustrating these changes. Also noteworthy are referee 1’s comments, which warn against misnomers that may cause confusion, emphasizing the need for metadata and the careful definition of what is a ‘warm’ or a ‘cold’ structure.

The dynamic shift is easier to validate if the same laboratory conducts the structure determinations at both low temperature (for best resolution) and high temperature (for thermal motion), and ideally on the same sample or from the same crystallization trial, thus eliminating possible experimental variances. This aspect expedites comparative descriptions such as those reported by Skaist Mehlman *et al.* (2023 ▸), where room-temperature crystallographic screens of the therapeutic target PTP1B using similar fragments were compared with cryo-structures. At the higher temperature, fewer ligands bound and often more weakly. However, a variety of temperature-dependent differences were observed, including unique binding poses, new binding sites and changes in solvation.

Interestingly, in our PDB survey we noted the number of low occupancies of bound waters that occur in many refinements, and the question arises as to whether this is a refinement concern or an aspect of temperature. This highlights, in any case, the need for the definition of a community standard of what is an acceptable occupancy or occupancy variation for bound waters, as the current modelling of water molecules still seems to be subjective. Reviews describing water binding exist (Levitt & Park, 1993 ▸), and the norm is to reject water molecules that have low occupancy, are located too far from the protein or have large ADPs or weak density peaks. However, a standard cutoff value for bound waters is typically adapted to data resolution and therefore is quite variable (Liebschner *et al.*, 2020 ▸).

## 3. Small-molecule ligands determined by chemical crystallography at 37°C and above

**Figure 1:**
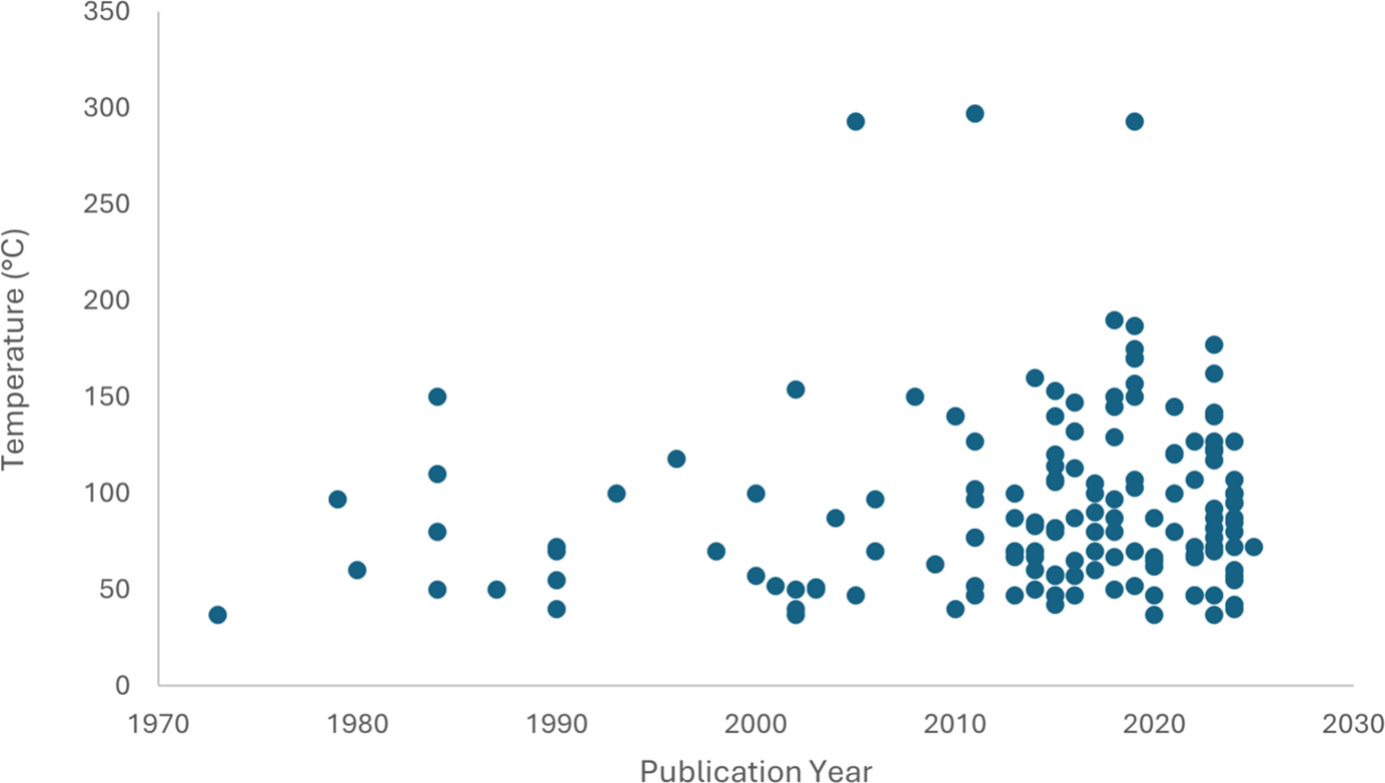
Plot of structures from the CSD Drug Subset obtained at temperatures of ≥37°C. Analysis was performed utilizing CSD version 6.00 in April 2025 with filter request: 3D coordinates determined.

**Figure 2:**
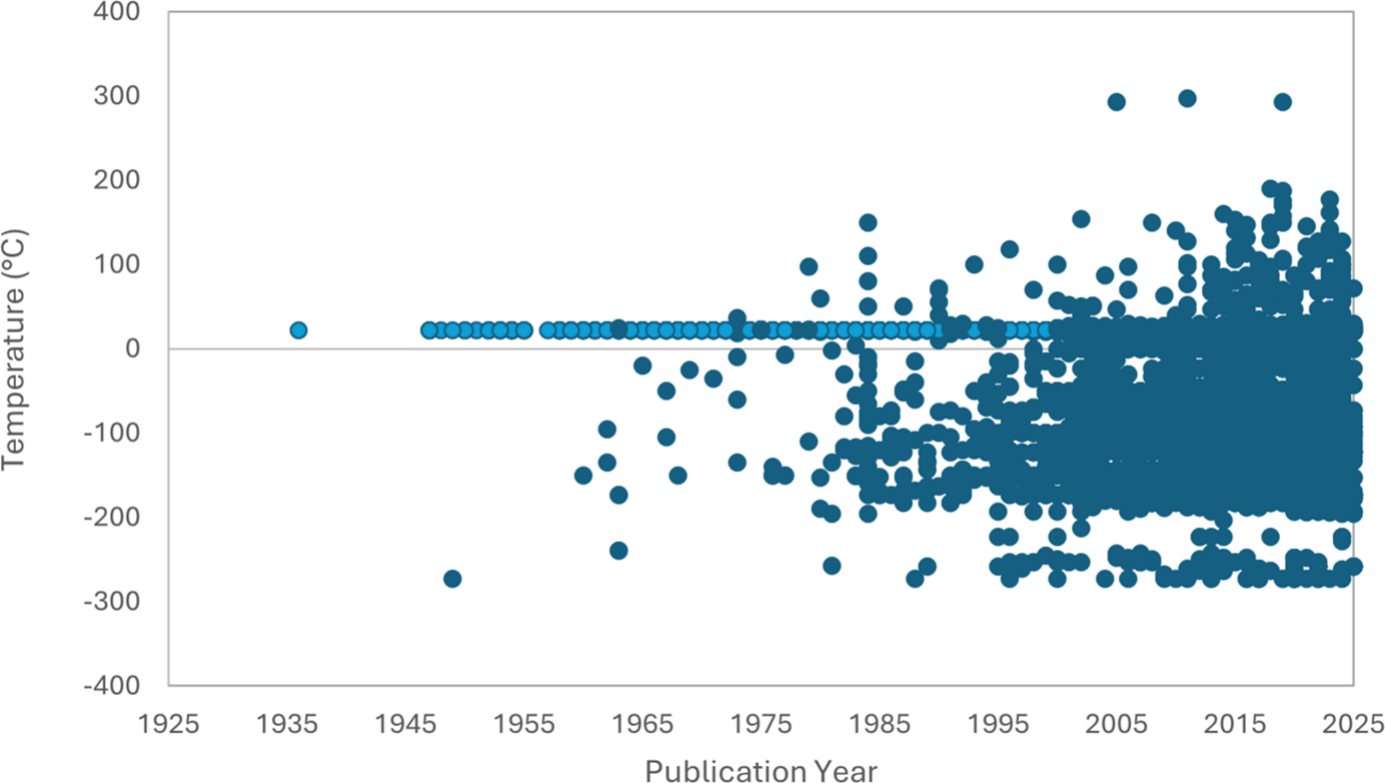
Plot of structures obtained from the CSD Drug Subset indicating all possible structures in the temperature analysis range −273 to 297°C. Data collected at 22°C (approximately room temperature) are indicated in light blue.

In addition to the PDB survey, we have also reviewed the Cambridge Structural Database (CSD) for structures determined at 37°C which are associated with drug development, some of which would be ligands or amino acids for receptor binding sites. The Cambridge Structural Database (Groom *et al.*, 2016 ▸) is a vital resource for small-molecule scientific research and at present contains over 1.4 million curated small-molecule entries. In this review, we utilized the CSD Drug Subset containing over 16 000 small-molecule structure entries for approved drug molecules (as defined by the DrugBank approved drug list; Wishart *et al.*, 2006 ▸). This includes organic compounds, hydrates, solvates, salt and metal complexes, where the temperature of structure analysis ranges from 0 to 570 K. This subset was searched for structures analysed at temperatures of ≥37°C (310 K), yielding approximately 159 hits at the date of analysis (Fig. 1 ▸). The full scope of temperature analysis for the entire CSD Drug Subset is significantly more vast (Fig. 2 ▸).

Assuming that both the ligand and protein conformation may change from a low to a high temperature, it is worth noting the interoperable data management when importing a ligand from the CSD utilizing *Coot* (Emsley *et al.*, 2010 ▸) for the consideration of conformations which may occur at variable temperatures. The structures specify the CIF data names and items such as cell_measurement_temperature requiring the author to specify the temperature in kelvin at which the unit-cell parameters were measured. Similarly, _diffrn_ambient_temperature indicates the mean temperature in kelvin at which the diffraction intensities were measured. The two items should ideally match. This is applicable for both small-molecule CIF and macromolecular CIF files. The small-molecule data available in the CSD report this value as ‘Temperature (K)’, the temperature at which the structure is obtained, and a search for such structures can easily be conducted. Importantly, this is not the temperature of synthesis or crystallization. When depositing data into the CSD, additional experimental notes for crystallization or chemical synthesis (relating to temperature, crystal-growth conditions *etc.*) can be provided as metadata; alternatively, the publication text must be reviewed. In light of understanding the total dynamics of systems under physiological conditions, the small-molecule community is encouraged to support this practice of reporting both the crystallization temperature as well as the diffraction temperature.

In contrast, when conducting a temperature analysis review of the macromolecular X-ray structures from the RCSB PDB, this differentiation is already a community practice. A temperature data search can be conducted by specifying Structure Attributes > X-ray Data Collection Details > Diffraction Source Temperature in the search criteria of the PDB, which allows a search for the mean temperature (in K) at which the diffraction intensities were measured, whereas Crystal properties > Temperature yields a search for the temperature (in K) at which a crystal was experimentally grown. These search parameters can be readily used when comparing the adopted conformations of macromolecular structures and bound ligands examined at variable temperatures.

**Figure 3:**
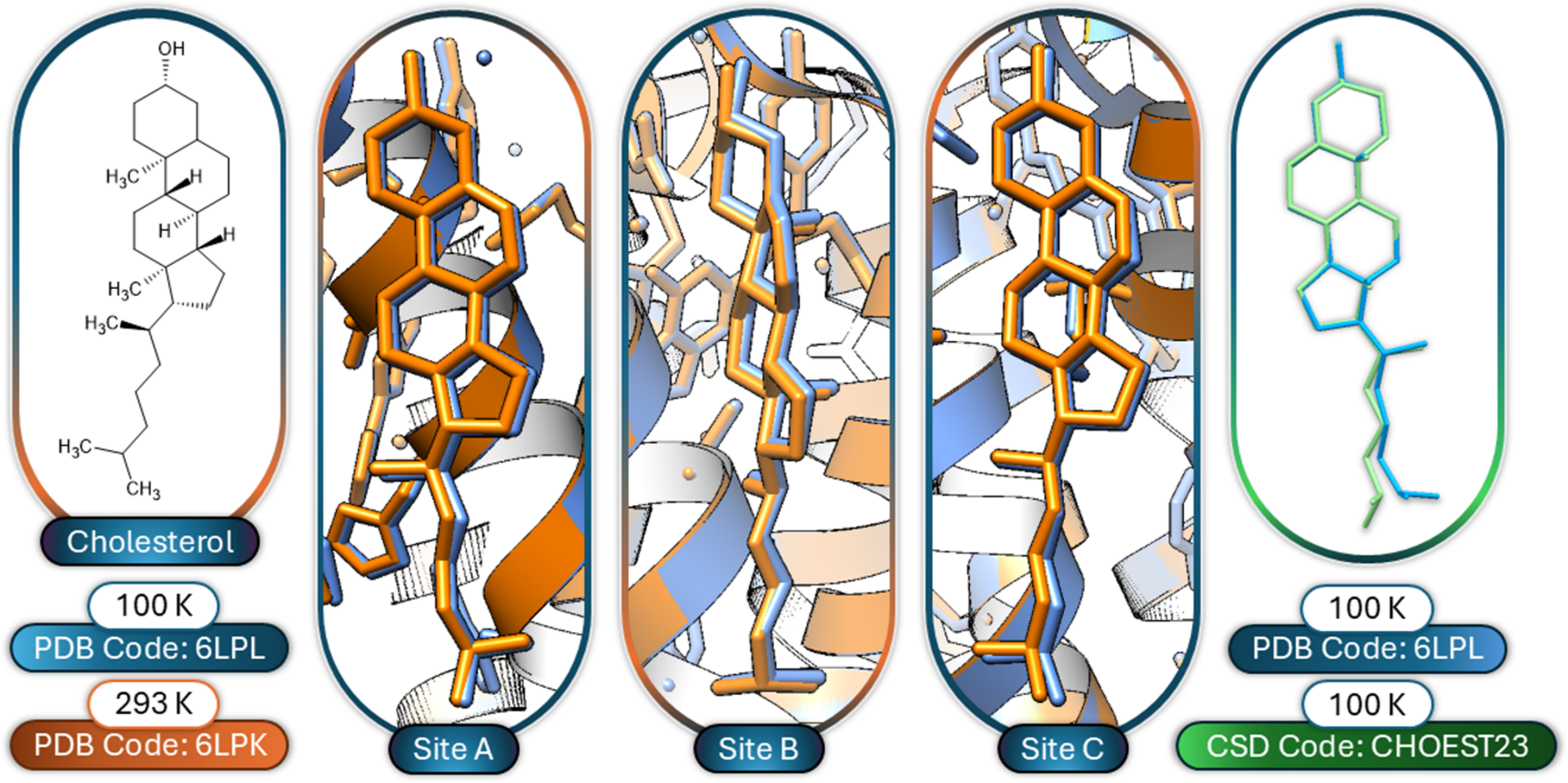
Line drawing of cholesterol with the three cholesterol sites in the adenosine A_2A_ protein. The blue structure was collected at 100 K (PDB entry 6lpl) and the orange structure at 293 K (20°C). The overlay of the structures shows the remarkable similarity despite the temperature change of nearly 200°C. The green and light blue overlay demonstrates the similarity of the 100 K small-molecule (CSD code CHOEST23) and macromolecular (PDB entry 6lpl, site B) structures.

Focusing on key amino-acid groups in the CSD Drug Subset, representative small-molecule examples analysed at 100 K and high temperatures were considered to identify whether temperature changes affected geometry. Examples such as cholesterol (CSD refcodes CHOEST23, CHOEST20, CHOEST24 and CHOEST21) are not markedly affected by temperature change when analysed between 100 K and 37°C. Some motion of the hydrocarbon tail is common, but it is unknown whether thermal motion or disorder is the cause (Hsu *et al.*, 2002 ▸). When analysing structures from the PDB, the consistency of the geometry of cholesterol is also notable in the crystallization of the adenosine A_2A_ membrane protein (Ihara *et al.*, 2020 ▸) with a crystallization matrix that contains cholesterol. The crystals, which were grown at 20°C (and were stable between 4 and 20°C), were analysed both by serial femtosecond crystallography at 20°C (and 4°C) and rotation synchrotron crystallography at 100 K. In both the 20°C and 100 K data sets the cholesterol interacts with the lipophilic (membrane-facing) sections of the protein at three sites. The three cholesterol molecules are in a near-identical orientation and position between the two structures (Ihara *et al.*, 2020 ▸), as depicted graphically in Fig. 3 ▸. The geometry of the small-molecule data (as seen in the CSD) and these cholesterol-containing macromolecules (found in the PDB) corroborate one another, with small motion of the tail.

**Figure 4:**
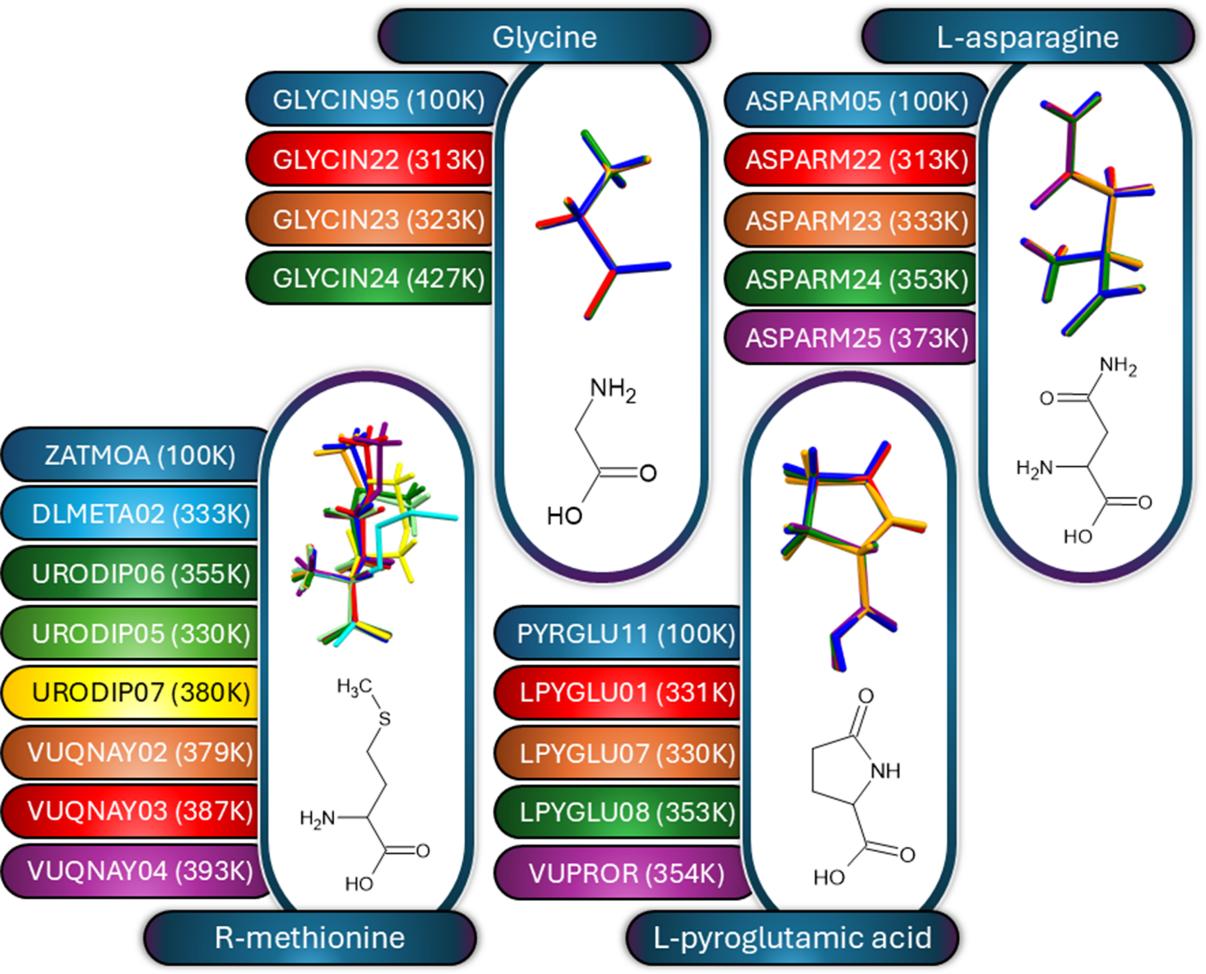
Line drawings of the four biologically relevant small-molecule structures obtained at high temperatures (≥37°C) as found in the CSD Drug Subset. Line drawings of the small molecules are shown in the insets of the respective overlays. The CSD codes and the data-collection temperature are also included. Nonbonding co-crystallites or solvent molecules without marked interactions were not illustrated in the overlay.

The CSD Drug Subset also contains small-molecule crystal data for biologically relevant amino-acid structures which have been collected at high temperatures (≥37°C), namely l-asparagine, glycine, l-pyroglutamic acid and *R*-methionine. The temperature-varied structures (diffraction data obtained at 100 K or higher) were overlaid and it was found that there were no marked deviations regardless of temperature, with the exception in the series being that *R*-methionine contained a longer aliphatic side chain. The overlays are shown in Fig. 4 ▸. Reasons other than temperature for the conformational changes in *R*-methionine were considered (see Supplementary Table S4). There are a number of variables indicated in the unit-cell parameters (variation in bond lengths, angles, volume and space group). While no solvent is present within the *R*-methionine structures, they do contain different co-molecules. Hydrogen interactions are only observed at the amine and carboxylic acid functional groups, leaving the tail without such interactions (only weak van der Waals inter­actions are observed). The flexible tail is therefore more free to vary, which may promote the different conformations. For the other molecules (glycine, l-pyroglutamic acid and l-asparagine) the variations in the unit cell are less pronounced. The crystal structures do not contain co-crystallites, although the asparagine structures do contain an aqua solvent molecule. These structures do indicate hydrogen interactions across the whole of the molecule, which may stabilize the particular conformation. Given that there are many variables at play, the exclusive effect of temperature on these crystal structures cannot yet be quantified, but it should be considered a variable that is worth considering in experimental design plans.

A PDB search for these specific compounds as standalone ligands yielded several macromolecular structures (203 entries for l-methionine, six entries for d-methionine, 438 entries for glycine, one entry for *R*-pyroglutamic acid, nine entries for *S*-pyroglutamic acid, 40 entries for l-asparagine and five entries for d-asparagine). None of these had been collected at both ‘high’ and ‘low’ temperatures for the same biological macromolecule by the same scientific investigators, and as such are not directly comparable without possible experimental variance. This highlights the need for high-temperature (∼37°C) and cryo (100 K) structural analysis to be conducted by the same study under identical experimental conditions in order to identify changes as clearly as possible.

## 4. Consistency of temperature: pre-crystallization through to and including X-ray diffraction data collection

**Figure 5:**
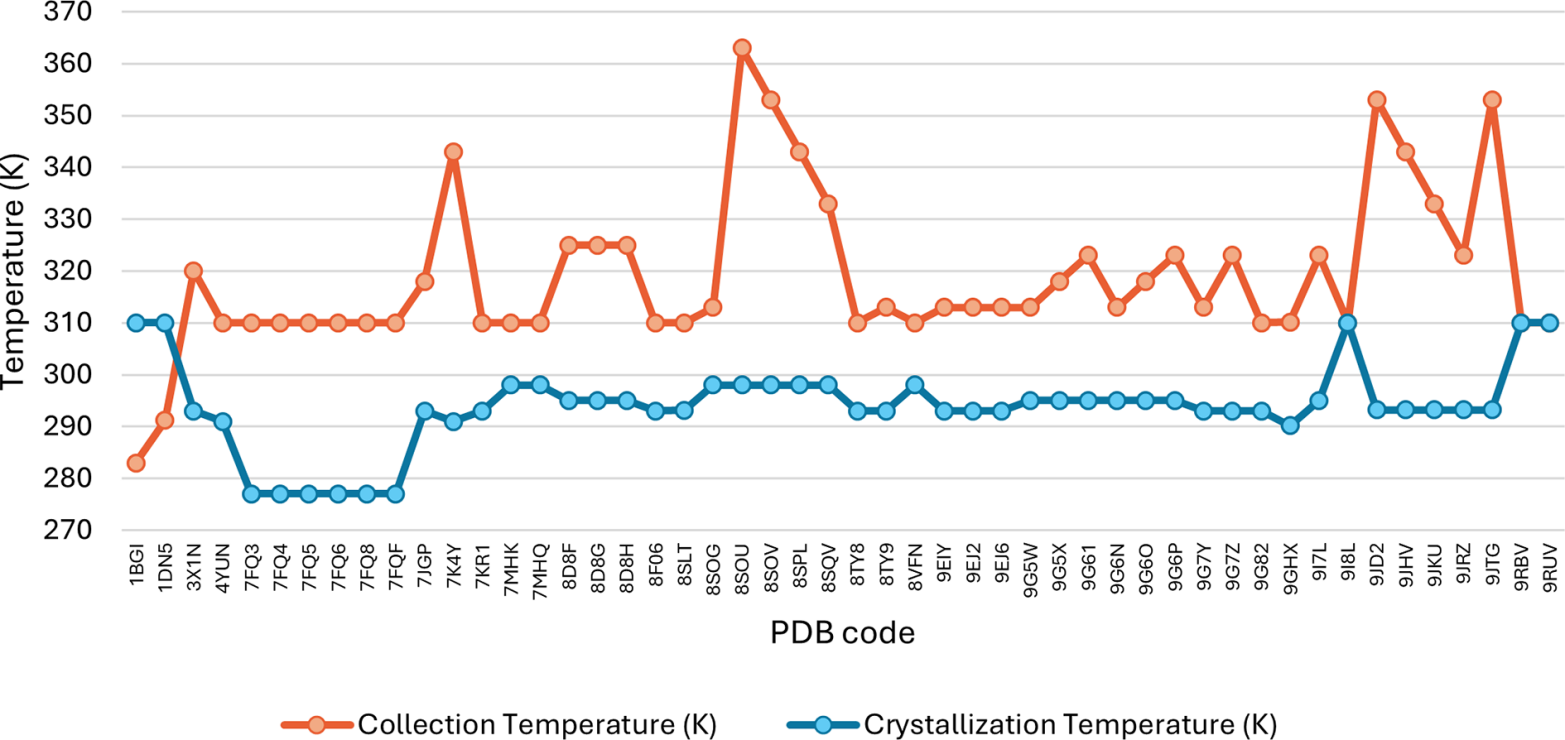
Line graph of the diffraction data-collection temperature (orange) of PDB structures that were determined at 310 K (*i.e.* 37°C) and greater, together with the respective crystallization temperature (blue). Data from relevant crystallization studies are also included. Metadata details are available in Supplementary Tables S1 and S2.

From the survey of available PDB structures, the macromolecular crystallography methods employed for realizing ≥37°C structures have yet to achieve consistency. Most striking is that very few have involved crystallization at these unusually high temperatures followed by analysis at the same temperature (Supplementary Tables S1 and S2). This is graphically illustrated in Fig. 5 ▸, which shows a plot of crystallization versus analysis temperature.

**Figure 6:**
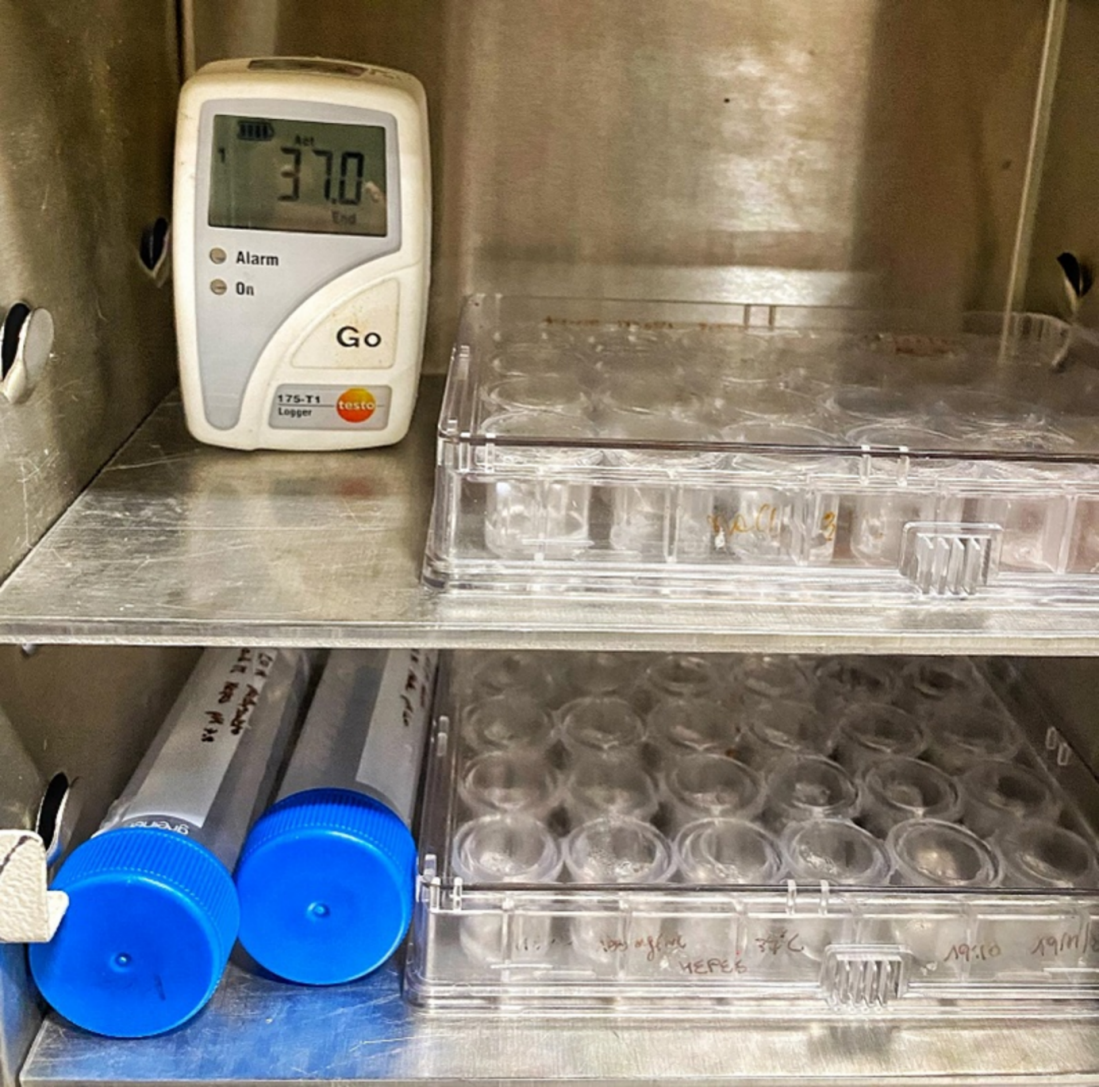
Photographs of the Linbro plate containing the bridges with the crystallization drops and of the solutions used to prepare crystals of lysozyme treated with [V^IV^O(acac)_2_] in an oven maintained at 37°C (Tito, Ferraro, Garribba *et al.*, 2025 ▸).

However, there are examples. Chevrier *et al.* (1986 ▸) studied the solvation of the left-handed oligonucleotide hexamer d(5BrC-G-5BrC-G-5BrC-G) in crystals grown at three temperatures: 5, 18 and 37°C. However, the diffraction data were not measured at 37°C despite one of their crystals having been grown at 37°C. Antonello Merlino’s group (Paolillo *et al.*, 2025 ▸; Tito, Ferraro, Garribba *et al.*, 2025 ▸; Tito, Ferraro & Merlino, 2025 ▸) achieved crystallization at 37°C with the use of an oven (see Fig. 6 ▸) and also carefully tried to preserve this temperature during transfer of their crystals to the diffracto­meter; they then performed diffraction data collection at 37°C.

The use of an oven is a simple and effective way to ensure temperature equilibrium. An alternative strategy for such experiments could be a room held at 37°C for the crystallization trays and crystal mounting before transfer to the diffractometer. This seems reasonably straightforward to achieve, requiring the simple adaptation of a home laboratory or a synchrotron hutch to be held at this temperature throughout.

## 5. Transfer to the diffractometer

The step that one can expect to be an Achilles heel of these studies above 37°C, cooling of the crystal to room temperature, would certainly occur if mounting and then transfer to the diffractometer were prolonged. A most extreme situation would be where the crystallization room were some distance from the X-ray laboratory, synchrotron hutch or neutron instrument, thus requiring courier transit in some form. A successful very short transfer time to the diffractometer was realized by Tito, Ferraro, Garribba *et al.* (2025 ▸), who remarked as follows:

Crystals of lysozyme treated at 37°C with [V^IV^O(acac)_2_] were fished with a loop and then inserted in a borosilicate glass capillary (glass 0500, linear absorption coefficient 71.0 µ cm^−1^) where droplets of the reservoir solution were previously inserted to reduce crystal dehydration. The capillary was maintained at 37°C before the crystal mounting. This procedure requires less than 30 s.

A heated laboratory held at 37°C is one option; however, housing the diffractometer may cause future problems. Modern diffractometers tend to protect instrument lifespan by ensuring that a high-temperature safety warning is activated. Thus, localized cooling of the detector in its immediate environs would need special care according to informal advice from diffractometer equipment manufacturers. Alternatively, a portable flexible incubator which can temporarily envelope the microscope or goniometer would be a possibly useful addition for such experiments to ensure consistent ‘climate control’.

During the collection of diffraction data the temperature has so far been controlled via the CryoStream apparatus from Oxford Cryosystems (https://oxcryo.com/products/cryostream/), which has a built-in heater device that achieves a range of crystal sample temperatures from 100 to 350 K. This device is very widely available, including at home X-ray laboratories, synchrotron beamlines and neutron macromolecular crystallography instruments.

## 6. Crystallization

One of the expected challenges of crystallization at 37°C or above is the increased solubility of the macromolecule; therefore, entering the nucleation zone of the crystallization phase diagram will require different conditions to crystallizing the macromolecule at a lower temperature, usually room temperature but also often 4°C, a typical cold-room setting. A standout very recent protein study, experiencing no obvious difficulty crystallizing at 37°C, is that of Paolillo *et al.* (2025 ▸), from which we quote.

For crystals grown at 20°C:

HEWL (13 mg ml^−1^) was crystallized using the hanging drop vapour diffusion method and 1.1 M sodium chloride and 0.1 M sodium acetate pH 4.0 as a reservoir. The reservoir solution had a volume of 500 µL, while the drop was 2 µL. Crystals formed within one day at 20°C.

For crystals grown at 37°C:

HEWL (100 mg ml^−1^) was crystallized at 37°C using the hanging drop vapour diffusion method under the same crystallization condition. Crystals were grown within a few hours. Pre-formed HEWL crystals were then exposed to stabilizing solutions containing the mother liquor saturated with Cs_2_[V^V^_2_O_4_(mal)_2_]·2H_2_O for a soaking time of a few hours at 37°C.

The obvious difference between the two sets of conditions is that the protein concentration was increased from 13 to 100 mg ml^−1^.

There is also a standout study involving the crystallization of a nucleic acid by Chevrier *et al.* (1986 ▸), from which we quote (with the permission of Elsevier):

Three-dimensional X-ray diffraction data were collected at 18°C on a Enraf–Nonius CAD4 diffractometer using the ω/2θ scan mode. The crystals grown at 5°C did not diffract X-rays but those grown at 18°C and 37°C did, albeit differently.

There are insufficient details of the crystallization conditions to be sure of the changes between the temperatures given by Chevrier *et al.* (1986 ▸), but we imagine that it is probably that the precipitating agent concentration was increased at the higher temperature:

Crystals were grown from solutions containing 20 mM sodium cacodylate (pH 6–5), 200 mM sodium chloride, and the oligomer at a concentration of 1 mg ml^−1^, by vapour diffusion at three temperatures, 5°C, 18°C and 37°C. The precipitating agent was 2-methyl-2,4-pentanediol at 10 to 60%.

The increased concentration is a common theme observed in crystallization performed at higher temperature.

As remarked in Section 1, there is also the classic crystallization work of Jollès & Berthou (1972 ▸) and their follow-up kinetic studies, as well as the PDB entry 1bgiX-ray crystal structure analysis study of Oki *et al.* (1999 ▸).

## 7. X-ray data collection

During X-ray data collection the risk of dehydration of the crystal has been reported, and suggestions on how to manage it can be found in the literature, varying from coating with hydrocarbon grease to the use of capillaries or sleeves or humidity-controlled devices, either home-built or those found at synchrotrons such as the T-REXX endstation (P14-2) at EMBL (see Tito, Ferraro, Garribba *et al.*, 2025 ▸; Jacobs *et al.*, 2024 ▸; Ebrahim *et al.*, 2022 ▸; Bowler *et al.*, 2015 ▸; Supplementary Table S1). The immediate concern about X-ray diffraction data collection, especially with extremely bright synchrotron beams, is enhanced X-ray radiation damage. This is a factor that has also been described for time-resolved serial synchrotron crystallography, and practical suggestions are available for the community considering time-resolved structure determination as a function of temperature (Schmidt *et al.*, 2012 ▸; Schulz *et al.*, 2022 ▸).

However, as rather simply remarked on by Tito, Ferraro, Garribba *et al.* (2025 ▸), as short an exposure time was used as possible:

X-ray diffraction data were collected at Elettra synchrotron on XRD2 beamline at 37°C. To reduce radiation damage 180 images of 1 degree were collected with an exposure time of 0.2 s using 1.000 Å radiation.

**Figure 7:**
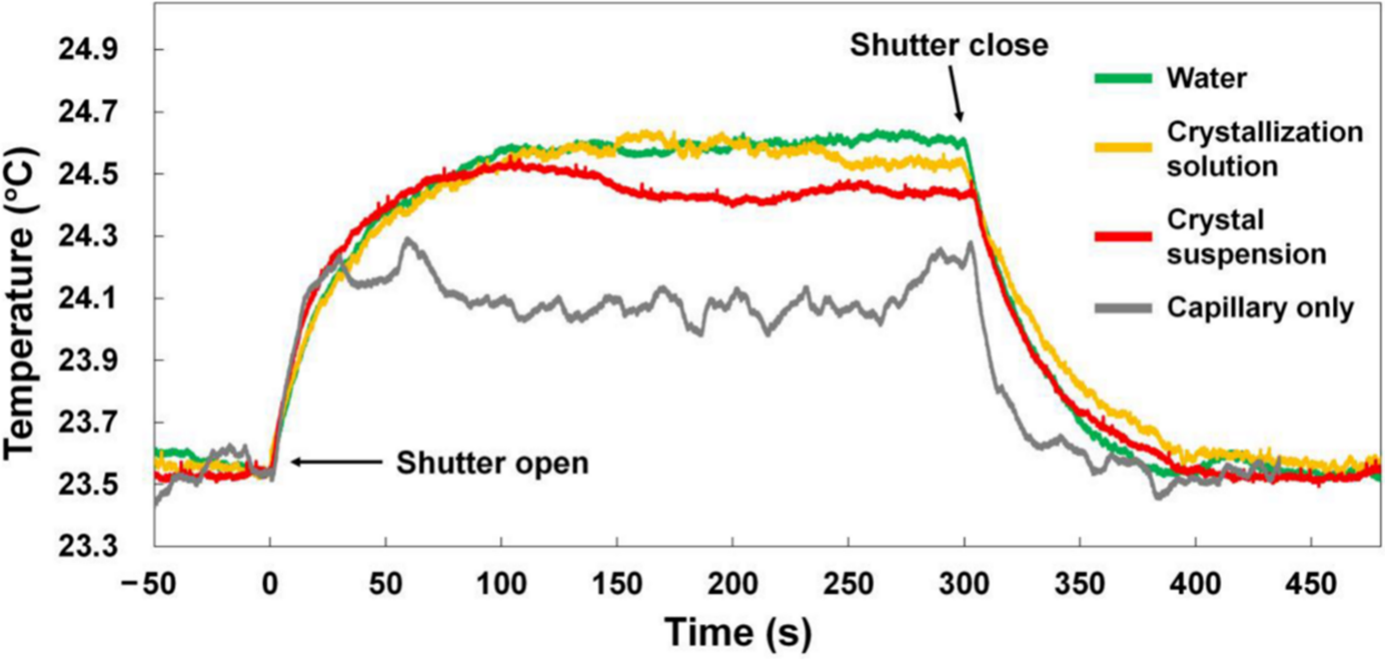
Two-dimensional profile of the temperature rise of a protein crystal due to X-ray exposure on the 7A beamline of the Pohang Light Source II, Republic of Korea from Kim & Nam (2023 ▸).

What about synchrotron X-ray beam heating of a crystal? Anything more than a 1°C increase would rather defeat the objective of the experiment of achieving a body-temperature crystal structure. There is by now a long history to considering this. To account for X-ray-induced heating within the crystals (Helliwell, 1984 ▸; Warren *et al.*, 2019 ▸), as a first step one can use an adiabatic heating model as a first approximation. Based on this model, which ignores any heat exchange with the environment of the crystal, the increase in temperature is given by the energy absorbed by the mass and the specific heat capacity of the sample. Such estimates are an upper boundary for a temperature-rise estimation, which do not take heat exchange with the environment into account. One must then model the thermal diffusion process into the surrounding regions using numerical simulation. It is important to note that appropriate simulations are highly demanding. However, Kuzay *et al.* (2001 ▸) made improved thermal models that included convection; temperature rises of 6 K were calculated for a cryocooled 100 µm thick crystal and of 18 K for a room-temperature air-cooled 1 mm thick crystal for an 8 keV 10^13^ photons s^−1^ mm^−2^ beam. Much smaller crystals than 1 mm will have much smaller temperature rises, but they may still possibly be significant. Also, different beamlines have different incident beam intensities. As a specific example, X-ray-induced heating experiments were performed on the 7A beamline of the Pohang Light Source II, Republic of Korea by Kim & Nam (2023 ▸). The X-ray energy and photon flux were 12 660 eV and 3 × 10^11^ photons s^−1^, respectively. The vertical and horizontal beam sizes of the X-rays were 100 and 300 µm (full-width at half-maximum; FWHM), respectively. The temperature of the sample (a quartz capillary filled with water, crystallization solution or lysozyme crystal suspension) gradually rose by 1°C every 100 s (see their Fig. 2 ▸*b* reproduced here as Fig. 7 ▸).

## 8. Protein crystal structures at temperatures well above 37°C and even up to 90°C

There are good-quality crystal structures from X-ray diffraction data measured at very high temperatures, *i.e.* up to 363 K (90°C), as documented in Supplementary Table S2. This means that the atomic order of thermophiles can be analysed under their physiologically relevant conditions. Presumably, the main hurdle will increasingly be X-ray radiation damage as these higher temperatures are explored. There may also be increasing anxieties regarding crystal stability or dehydration. A potential alternative for high-temperature X-ray macromolecular crystallography is the use of neutron diffraction macromolecular crystallography (nMX). It faces similar challenges, such as crystal dehydration, with the long measurement periods required for nMX being the primary concern, and so stability at that high temperature would be needed over potentially two weeks. However, nMX would have the marked advantage of no radiation damage and, at the very least, the lessons learnt by nMX in crystal handling can be applied in high-temperature X-ray crystallography. There are currently no entries in the PDB for nMX structures at temperatures of 37°C or above. At this time there is no widely used method for obtaining >80°C macromolecular data sets other than those already described for 37°C data collections.

## 9. Conclusions and avenues for future development

It is clearly feasible to obtain crystal structures at 37°C and across a wide range of biological macromolecules by managing conditions to ensure uniformity of potential equilibria, such as crystallization conditions ranging from standard low, *i.e.* near room temperature (to encourage nucleation), to higher temperatures (∼37°C) that may require increased protein concentrations. Ensuring temperature consistency during crystal growth, mounting and transfer to the diffractometer either with the use of an oven, a heated room or an incubated hutch would be good. Diffraction data collection at 37°C can be easily controlled with the use of Cryostreams and the use of a capillary or grease to prevent dehydration at the higher temperature. It should be feasible for an experimental setup at a synchrotron beamline to perform data collection at 100 K to secure the highest resolution detail structure and then immediately perform data collection at 37°C. If a ligand remains bound at body temperature, then it offers a practical filter of hits that may be more relevant for drug development, as structural stability is then accounted for. It is also feasible to obtain macromolecular crystal structures up to 90°C, although here there are far fewer examples than those studied at 37°C.

In addition to temperature considerations, the effect of inter-operator variability should be taken into account. To eliminate any human/laboratory variation factor, and to ensure that possible changes in any determined 3D macromolecular structure between temperatures are in fact significant, data collection of crystals at 100 K followed by 37°C is encouraged to be conducted by the same laboratory and, if possible, using the same batch of crystals.

This consideration for experimental consistency could be extended further. Due to the increased dynamics and foreseeing the relevance of temperature effects in structural drug development, the small-molecule community is encouraged to record the metadata of crystal-growth temperature as well as those of diffraction data collection to match the metadata protocols used by the macromolecular community.

The methods employed for successful high-temperature macromolecular crystallography vary considerably. It seems clear that the ideal is crystallization, transfer of crystal to the diffractometer and measurements on the diffractometer all being performed at the sought-after temperature of 37°C or above. This ideal has nearly been reached for 37°C in that the only step of vulnerability was that of transfer of the crystal to the diffractometer, but in one example this was kept to a maximum time of 30 s (Tito, Ferraro, Garribba *et al.*, 2025 ▸). From the database surveys, no obvious trends in either the PDB or CSD are identifiable as yet. Some structures do show variation, while others are consistent across a temperature range, for both macromolecules and small molecules. It would be inappropriate to assume that temperature dynamics in protein structures is possible at 37°C but small molecules will always behave consistently at any temperature. Individual chemical properties, packing and crystallization energies may affect conformations. Hence, there is a need for systematic studies (100 K and 37°C for both proteins and small molecules) to be encouraged. Quite simply, we need many more examples of 37°C structural studies. This would provide increased clarity on whether structures change at 100 K compared with when determined at 37°C. Perhaps some things change more than others. Ligands with long side chains or amino-acid side chains that are long may show more flexibility at 37°C, whereas short side chains have limited scope for more flexibility. Also, a much better level of evidence is needed for what bound waters are doing at 37°C versus other, lower, temperatures. At present it is not possible to determine whether it is generally true that all noncovalent ligands bind less well at 37°C versus 100 K. It is also not known whether different types of ligands (*i.e.* metalated ligands versus organic based ligands) will indicate variable binding.

Also, new protein crystal structures should ideally have their raw diffraction data available. These being available would allow more consistent checks of the diffraction resolution limit according to paired model refinement (Malý *et al.*, 2020 ▸) and show up cases of unusually pronounced diffuse scattering. Also, the availability of raw diffraction data would be beneficial for the reworking of their processing as software improvements dramatically increase the refinement of data. These advantages will also fuel the use of AI without author bias or outdated models affecting the analysis.

Overall, our topical review reveals a few hurdles to be overcome but many successes in reaching patient-relevant and thermophile-relevant structures.

*Note added in proof*. Doukov *et al.* (2025 ▸) report observations on rubredoxin crystal structures studied up to 120°C (PDB entry 9y2y at 393 K).

## 10. Related literature

The following references are cited in the supporting information for this article: de Sá Ribeiro & Lima (2023 ▸), Doukov *et al.* (2023 ▸), Du *et al.* (2023 ▸), Fukuda & Inoue (2015 ▸), Greisman *et al.* (2024 ▸), Guerrero *et al.* (2023 ▸), Keedy *et al.* (2015 ▸), Otten *et al.* (2020 ▸), Parkins *et al.* (2024 ▸), Sammons *et al.* (2019 ▸) and Schuller *et al.* (2021 ▸).

## Supplementary Material

Supplementary Tables. DOI: 10.1107/S2059798325010617/nw5135sup1.pdf
