# Supplementary material for: Towards patient-relevant structures: reviewing body-temperature biological macromolecules and their ligands for pharmaceutical applications
Source: Acta Crystallogr D Struct Biol. 2026 Jan 1;82(Pt 1):1–10. doi: 10.1107/S2059798325010617 (PMC12809522; doi:10.1107/S2059798325010617)
Supplement: Supplementary file 1 [file d-82-00001-sup1.pdf]

Acta Cryst D
STRUCTURAL
BIOLOGY

Volume 81 (2025)

Supporting information for article:

Towards patient-relevant structures: reviewing body-temperature biological macromolecules and their ligands for pharmaceutical applications

Alice Brink, John R. Helliwell and Francois J. F. Jacobs

Table S1 Macromolecular crystal structures deposited in the PDB for 37°C (310K).

PDB code	Crystallization Temperature	Collection Temperature	Key crystallization conditions	Resolution (Å)<br><br>R and R_{free} (%);<br><br><I/σI> from PDB Report;<br><I/σI> from authors' publication, overall (high resolution shell).;<br><br>$CC_{1/2}$ overall (high resolution shell).	Space group	Cell para-meters (Å)	Additional notes on the PDB model from PDB REDO refinement.	Additional notes on the publication and raw data.	Ref.
1BGI	310K	283K	Crystallization was performed by the batch method at 310K. The same way as described by Jolles and Berthou (1972)., pH 4.7, batch method	1.7 Å<br><br>0.186, 0.216<br><br>0.97 (at 1.67Å).<br><br>-	$P2_12_12_1$	56.44<br>73.73<br>30.43	PDB REDO removed 42 of the deposited bound waters out of an initial total of 108.		Oki et al. (1999)
1DN5	5°C, 18°C, and 37°C (310 K)	18.15 °C (291.15K)	*"The crystals grown at 5°C did not diffract X-rays but those grown at 18°C and 37°C did, albeit differently."*	1.40<br><br>12.5, 15.3<br><br>0.91 (at 1.38Å);<br>Not given but instead authors quote 25% of intensity data were>2σ(I).	$P2_12_12_1$	17.93<br>30.83<br>44.73	PDB REDO removed 47 of the deposited bound waters out of an initial total of 82. In the RT model 1DN4 21 waters removed by PDB REDO out of an initial total of 60.	The main finding of the paper was stated as follows:- "*The main difference resides in a more extensive hydration shell in the crystal grown at high temperature than in the crystal grown at low temperature*."<br><br>Important to note that since PDB REDO made each model have very similar numbers of bound waters (35 versus 39), and viewing the structures in Coot suggests that this main finding of the effect may be artificially removed using PDB REDO	Chevrier et al. (1986)

4YUN	291K	310K	Crystals were grown by mixing equal volumes of well solution (100 mm HEPES pH 7.5, 23% PEG 3350, 5 mM TCEP) and protein (60 mg/ml in 20 mM HEPES pH7.5, 100 mM NaCl, 0.5 mM TCEP) in the hanging-drop format	1.58<br><br>0.117, 0.150.<br><br>1.22 (at 1.58Å);<br>13.26 (1.45).<br><br>1.00 (0.56).	$P2_12_12_1$	42.85<br>52.58<br>89.41	9 bound waters removed out of 106 in the 4YUN deposition.	Multi-conformer synchrotron model of CypA at 310 K	Keedy et al. (2015)
7FQ3	277K	310K	20 mM imadazole (pH 5.4-5.8), 16-21% PEG 400, 125 mM $MnCl_2$, pH 5.6, vapor diffusion, sitting drop,	1.30<br><br>0.152, 0.187.<br><br>0.25 (at 1.30Å);<br>15.4 (0.4).<br><br>0.997 (0.360).	$P2_12_12_1$	34.18<br>45.49<br>99.33	75 bound waters removed by PDB REDO, including a few alternate location bound waters out of a deposited file. Total of 163 waters including alternates.		Greisman et al. (2024)
7FQ4	277K	310K	20 mM Imadazole (pH 5.4-5.8), 16-21% PEG 400, 125 mM $MnCl_2$, pH 5.6, vapor diffusion, sitting drop,	1.33<br><br>0.146, 0.186.<br><br>0.47 (at 1.32Å);<br>24.5 (0.6).<br><br>0.999 (0.301).	$P2_12_12_1$	34.15<br>45.23<br>99.22	41 bound waters removed by PDB REDO, including a few alternate location bound waters, out of a deposited file total of 163 waters including alternates.	Possible evidence of radiation damage.	Greisman et al. (2024)
7FQ5	277K	310K	20 mM Imadazole (pH 5.4-5.8), 16-21% PEG 400, 125 mM $MnCl_2$, pH 5.6	1.35<br><br>0.150, 0.190.<br><br>0.34 (at 1.35Å);<br>21.3 (0.5).	$P2_12_12_1$	34.19<br>45.30<br>99.25	73 bound waters removed by PDB REDO, including a few alternate location bound waters, out of a deposited file total of 163 waters including alternates.		Greisman et al. (2024)

				0.999(0.328).					
7FQ6	277K	310K	20 mM Imadazole (pH 5.4-5.8), 16-21% PEG 400, 125 mM $MnCl_2$, pH 5.6	1.29<br><br>0.140, 0.179.<br><br>0.37 (at 1.29Å).<br>31.6 (0.6).<br><br>1.000(0.326).	$P2_12_12_1$	34.18<br>45.30<br>99.25	60 bound waters removed by PDB REDO, including a few alternate location bound waters, out of a deposited file total of 162 waters including alternates.	Possible evidence of radiation damage.	Greisman et al. (2024)
7FQ8	277K	310K	20 mM Imadazole (pH 5.4-5.8), 16-21% PEG 400, 125 mM $MnCl_2$, pH 5.6.	1.21<br><br>0.126, 0.164.<br><br>0.30 (at 1.21Å);<br>14.1 (0.4).<br><br>0.999(0.380).	$P2_12_12_1$	34.29<br>45.65<br>99.03	74 bound waters removed by PDB REDO, including a few alternate location bound waters, out of a deposited file total of 164 waters including alternates.		Greisman et al. (2024)
7FQF	277K	310K	20 mM Imadazole (pH 5.4-5.8), 16-21% PEG 400, 125 mM $MnCl_2$, pH 5.6.	1.23<br><br>0.131, 0.169.<br><br>0.31 (at 1.23Å);<br>12.3 (0.3).<br><br>0.999(0.321).	$P2_12_12_1$	34.30<br>45.71<br>99.12	76 bound waters removed by PDB REDO, including a few alternate location bound waters, out of a deposited file total of 163 waters including alternates.		Greisman et al. (2024)
7KR1	293K	310K	100 mM TRIS, pH 8.5, 100 mM Sodium Acetate, 28% PEG4000.	1.55<br><br>0.180, 0.221.<br><br>1.02 (at1.55Å).<br>9.07 (0.51)<br><br>0.999 (0.399)	C2	134.84<br>30.45<br>37.88<br><br>98.95◦	One by PDB REDO (out of 18) supporting Fo-Fc density for removed water.		Schuller et al. (2021)

7MHK	298K	310K	22% PEG 4000, 100 mM HEPES pH 7.0, 3<br>-5% DMSO,	1.96<br><br>0.198, 0.247.<br><br>1.05 (at 1.97Å);<br>5.4(0.3).<br><br>0.990(0.352)	C2	114.30<br>54.29<br>44.97<br><br>102.12◦	37 bound waters removed by PDB REDO		Ebrahim et al. (2022)
7MHQ	298K	310K	22% PEG 4000, 100 mM HEPES pH 7.0, 3-<br>-5% DMSO,	1.96<br><br>17.1, 23.5.<br>5.3964 (0.3216)<br><br>0.55 (at 1.97Å);<br>0.9902 (0.3522)	C2	114.30<br>54.29<br>44.97<br><br>102.12◦	N/A	Not found in PDB-REDO: *Multi-model refinement*. Ensemble X-ray structure. No supporting information available for review. Values partly obtained from PDB file.	Ebrahim et al. (2022)
8F06	293K	310K	2 µL 1 M ammonium sulfate + 2 µL 10 mg/ml proteinase K in 50 mM TRIS, pH 7.5	1.80<br><br>0.116, 0.147.<br><br>2.91 (at1.81Å).<<Strong data;<br>25.6 (3.7). <<Strong data;<br><br>0.998(0.886). <<Strong data;	$P4_32_12$	67.95<br>102.39	65 bound waters removed by PDB REDO out of 239.<br>3 of these are in the difference map peaks list above 5σ.		Doukov et al. (2023)
8SLT	293.15K	310K	30% PEG 3350, 200 mM Li_2SO_4, 100 mM<br>bis-TRIS pH 5.5,	1.96<br><br>0.174, 0.206.<br><br>1.09 (at 1.95Å);<br>7.79 (0.63).	$P2_12_12_1$	39.98<br>64.49<br>137.21	12 bound waters removed by PDB REDO out of 68.<br>4 of these bound waters could be reinstated into PDB REDO Fo-Fc peaks.		Guerrero et al. (2023)

				0.996(0.358).					
8TY8	293K	310K	200 mM Na acetate pH 3.6 100 mM TRIS pH 8.5 20% PEG 3350 1 mM DTT.	1.40<br><br>0.182, 0.206.<br>10.7 (N/A)<br><br>1.51(at 1.40Å).<br><br>0.999 (0.355)	$P2_12_12_1$	39.42<br>41.81<br>92.09	24 bound waters removed by PDB REDO out of 115. One bound water could be reinstated based on a PDB REDO Fo-Fc map peak.	Unpublished results. Values obtained from available PDB file.	Deck et al. To be published
8TY9	293K	313K	200 M Na acetate pH 3.6 100 mM TRIS pH 8.5 20% PEG 3350 1 mM DTT,	1.43<br><br>0.192, 0.213.<br>6.6 (N/A)<br><br><br>1.49 (at 1.43Å).<br>0.997 (0.404)	$P2_12_12_1$	39.42<br>41.74<br>92.14	23 bound waters removed by PDB REDO out of 114.		Deck et al. To be published
8VFN	298K	310K	24-30% PEG 4000, 0.1 M sodium citrate (pH 5.6-6.0), 0.2 M ammonium acetate.	1.29<br><br>0.159, 0.167.<br>1.43 (at 1.29Å).<br><br>11.4 (0.5)<br>0.999 (0.329)	P3	84.29<br>84.29<br>41.06	63 bound waters removed by PDB REDO out of 231.		Parkins et al. (2024)
9GHX	290.15K	310.15K	HEWL, ReAA, imidazole, 1.0 M NaCl, and 0.05 M sodium acetate at 4.5 pH.	2.20<br><br>0.204, 0.271.<br><br>1.13 (at 2.18Å);<br>5.2 (0.8).<br><br>0.985 (0.434).	$P4_32_12$	81.49<br>81.49<br>37.41	1 bound water removed by PDB REDO out of 26.	Raw diffraction images underpinning 9GHX are available at Zenodo:- https://zenodo.org/records/13331546	Jacobs et al. (2024)
9EIY	293K (room temperature)	313 K	0.1 M HEPES pH 7.5, 0.5 µL of 100 mM	2.22	$P2_1$	45.89<br>120.17	11 bound waters removed by PDB REDO out of 17.	Inconsistent stats for <I/σ> possible described by	McLeod et al. (2025)

			$MnCl_2$, 100 mM GDP, and 18-28% PEG 3350, vapor diffusion, hanging drop.	0.213, 0.255.<br>1.00 (at 2.22Å).<br><br>3.3 (0.1)<br>0.989 (0.603)		61.41<br><br>110.19◦	Two could be reinstated based on the PDB REDO's Fo-Fc map peaks.	CCP4 Truncate log file stating "*Processed data contains a lot of weak data at high resolution.*"	
9EJ2	293K (room temperature)	313 K	0.1 M HEPES pH 7.5, 0.5 µL of 100 mM $MnCl_2$, 100 mM GDP, and 18-28% PEG 3350, vapor diffusion, hanging drop.	2.11<br><br>0.1890, 0.2317<br>1.01 (at 2.12Å)<br>6.2 (0.2)<br><br>0.996 (0.614)					McLeod et al. (2025)
9EJ6	293K (room temperature)	313 K	0.1 M HEPES pH 7.5, 0.5 µL of 100 mM $MnCl_2$, 100 mM GDP, and 18-28% PEG 3350, vapor diffusion, hanging drop.	2.40<br><br>0.1997, 0.2250<br><br>4.0 (0.8)<br>1.98 (at 2.40Å)<br><br>0.985 (0.606)					McLeod et al. (2025)
9G5W	295K	313K	35% (w/v) PEG 3350, 200 mM $LiSO_4$ and 10 mM HEPES/NaOH, PH 7.5.	1.70Å<br><br>0.173 , 0.207.<br><br>1.23 (at 1.70Å).<br>3.59 (0.98).<br><br>92.27(38.03).	I222	94.20<br>103.05<br>99.25	74 waters removed out of 310. 5 could be reinstated into the PDB REDO Fo-Fc peaks list.	Key comments in the rebuttal to referee 2: "*We clearly demonstrate new results that critically depend on the implementation of 5D-SSX, namely demonstrating in a structural context that reaction turnover is directly linked to temperature, and that reaction key intermediates can only be determined at these elevated temperatures (Fig. 4).*"<br><br>Another point of referee 2: "*The authors' choice of temperature domain from – 10 °C to 70 °C is appropriate for the goals of the method*".	Schulz et al. (2025)

								A third point in reply to referee 2: "*at 20 °C there is almost no change in the closed ring structure, while at 50 °C, the majority of the density can be explained by an open ring intermediate. These data unambiguously demonstrate that structural intermediates can be resolved that are not visible (at high occupancy) at ambient temperatures*".	
9G6N	295K	313K	(35% (w/v) PEG 3350, 200 mM $LiSO_4$ and 10 mM HEPES/NaOH, PH 7.5)	1.70Å<br><br>0.160, 0.200.<br><br>1.56 (at 1.70Å).<br>4.42(1.65).<br><br>93.27(62.3).	I222	94.20<br>103.05<br>99.25	53 waters removed out of 280 by PDB REDO. 7 could be reinstated into the PDB REDO Fo-Fc peaks list.	Xylose Isomerase collected at 40°C using time-resolved serial synchrotron crystallography with glucose at 60 seconds	Schulz et al. (2025)
9G7Y	293K	313K	CTX-M-14 solution (22 mg/ml) was mixed with 45% precipitant solution (40% PEG8000, 200 mM lithium sulfate, 100 mM sodium acetate, pH 4.5) and with 5% undiluted seed stock in batch crystallization setups.	1.70Å<br><br>0.155, 0.178.<br><br>1.66 (at 1.70Å).<br>7.02(1.71).<br><br>0.978(0.714).	P 3_221	42.32<br>42.34<br>234.84	44 waters removed out of 146 by PDB REDO. 4 could be reinstated into the PDB REDO Fo-Fc peaks list.		Schulz et al. (2025)
9G82	293K	310K	CTX-M-14 solution (22 mg/ml) was mixed with 45% precipitant solution (40% PEG8000, 200mM lithium sulfate, 100mM	1.70<br><br>0.169, 0.200.<br><br>1.09 (at 1.70Å).	P 3_221	42.25,<br>42.25,<br>234.85	69 waters removed out of 172. 3 could be reinstated into the PDB REDO Fo-Fc peaks list.		Schulz et al. (2025)

			sodium acetate, pH 4.5) and with 5% undiluted seed stock in batch crystallization setups.	4.36 (0.66).<br><br>0.959(0.252).					
9I8L	37°C	310K	Crystals of the protein were obtained using a sitting drop vapour diffusion method and drops of two microliters. The crystallization drops were set up by mixing lysozyme (concentration 100 mg mL^{-1}, 37°C temperature) with reservoir solution (1.1 M NaCl, 0.1 M sodium acetate at pH 4.0, 37°C). Linbro plates containing the drops were maintained at 37°C within a stove.<br><br>Crystals of lysozyme treated at 37°C with $[V^{IV}O(acac)_2]$ were fished with a loop and then inserted in a borosilicate glass capillary (glass 0500, linear absorption coefficient 71.0 µ cm^{-1}) where droplets of the reservoir solution were previously inserted to	1.83<br><br>0.200, 0.284.<br><br>1.21 (at 1.83Å).<br>8.9 (0.3).<br><br>0.997 (0.369).	P 4_32_12	80.86<br>36.83	No PDB REDO available.		Tito, Ferraro, Garribba et al. (2025a)

			reduce crystal dehydration. The capillary was maintained at 37°C before the crystal mounting. This procedure requires less than 30 seconds. X-ray diffraction data were collected at Elettra synchrotron on XRD2 beam line at 37°C.						
9RBV	37 °C and 20 °C.	310K	HEWL (100 mg mL^{-1}) was crystallized at 37 °C using the hanging drop vapour diffusion method under the same crystallization condition*. Crystals were grown within a few hours. Pre-formed HEWL crystals were then exposed to stabilizing solutions containing the mother liquor saturated with $Cs_2[V^V{}_2O_4(mal)_2]\cdot 2H_2O$ for a soaking time of a few hours at 37 °C. X-ray diffraction data were collected from one crystal at the XRD2 Beamline of the Elettra synchrotron (Trieste, Italy) at 37 °C. * HEWL (13 mg mL^{-1}) was	2.09<br><br>0.213, 0.293.<br>1.63 (at 2.08Å)<br>8.2 (0.9).<br>0.997 (0.323).	P 4_32_12	79.10<br>37.40	No PDB REDO available.	Key results: Crystallographic data reveal that, when protein crystals are incubated with the V compound at room temperature (25 °C) and at pH 4.0, $[V^{IV}O]^{2+}$, $[V^V{}_2O_5(mal)]^{2-}$, $[V^V{}_{10}O_{26}]^{2-}$ and $[V^V{}_{10}O_{28}]^{6-}$ are bound to the protein, while at 37 °C, under the same conditions, only $[V^{IV}O]^{2+}$ interacts with HEWL. $[V^V{}_{10}O_{28}]^{6-}$ can bind the protein both covalently (as $[V^V{}_{10}O_{26}]^{2-}$ ion) and non-covalently. Whereas the transformation of $[V^V{}_2O_4(mal)_2]^{2-}$ to $[V^V{}_2O_5(mal)]^{2-}$ is expected on the basis of thermodynamic considerations, the formation of V_{10} and of the V_{10}–HEWL adduct is not easily predictable.	Paolillo et al. (2025)

			crystallized using the hanging drop vapor diffusion method and 1.1 M sodium chloride and 0.1 M sodium acetate pH 4.0 as a reservoir. The reservoir solution had a volume of 500 µL, while the drop was 2 µL. Crystals formed within one day at 20 °C.						
9RUV	37 ◦C	310K	Crystals of Rh/HEWL adducts were obtained at 37 ◦C, treating crystals of the metal-free protein grown at 37 ◦C in 0.010 M HEPES pH 7.5 & 2.00 M sodium formate with a solution of the reservoir containing a large excess of $[Rh_2(\mu\text{-}O_2CCH_3)_4]$ for 1 day.	2.12<br><br>0.198, 0.261<br><br>1.63 (at 2.12Å)<br>13.2 (0.9)<br><br>0.998 (0.405)	$P\ 4_32_12$	80.43<br>36.14		Key descriptions from the abstract:-<br>The structures of the Rh/HEWL adduct formed at 20 ◦C (obtained from data collected at 100 K) and at 37 ◦C under the same experimental conditions are very similar, with metal binding sites that are conserved. However, metal-containing fragment occupancy is higher in the structure obtained at 37 ◦C, suggesting a role of temperature in defining the protein metalation process.	Tito, Ferraro, & Merlino (2025b)

Footnote: Relevant data with regards to high temperature collection or crystallization are also included.

Table S2 Macromolecular crystal structures deposited in the PDB for data well above 37°C (310K).

PDB code	Crystallisation Temperature	Collection Temperature	Key crystallisation conditions	Resolution (Å)<br><br>R and R_{free}<br><br><I/σI> from PDB Report;<br><I/σI> from authors' publication, overall (high resolution shell).;<br><br>$CC_{1/2}$ overall (high resolution shell).	Space group	Cell parameters (Å)	PDB Validation report assessment (Clash score; the authors own specific comments of interest based on the PDB report).	Comments	Additional Comments	Ref.
3X1N	293K	320K	5.5% PEG 4000, 0.1M sodium acetate, 0.075M copper sulphate, pH 4.5,	1.55Å<br><br>0.115, 0.131<br><br>22.1 (2.5)<br>2.41 (at 1.55Å);<br><br>0.846	H3	116.30<br>116.30<br>85.65	40 bound waters removed by PDB REDO out of 184 bound waters. 30 bound waters could be reinstated though in terms of fitting into an Fo-Fc PDB REDO peak above 5σ being seen.			Fukuda & Inoue (2015)
6NBS	295 K	446.15 K Suggested error in text, see right.	33% PEG 5,000 MME, 0.25 M ammonium sulfate, 0.1 M MES, pH 6.5	1.90<br><br>0.1801, 0.2218	$P12_11$	48.895<br>71.242<br>60.167		Droplets were allowed to equilibrate over the reservoir	X-ray diffraction data were collected at Advance Photon	Sammons et al. (2019)

		Publication text indicates collection temperature is -173 °C		2.46 (at 1.89Å)<br>35.1 (5.02)<br><br>$CC_{1/2}$ not reported				solution at room temperature (**approximately 22 °C**).	Source (APS) beamline 21-ID-F at **−173 °C** using a wavelength of 0.9798 Å and a MarMosaic225 CCD detector.	
7JGP	293K	318K	500 µL total volume: 50 mM<br>CHES (pH 9.5), 150 mM NaCl, 0.474 mM NiCl2, 12.6% PEP sitting drop: 7.6 µL reservoir, 2 µL OF 25 µM ferritin, 2.4 µL OF 5 nM<br>H_2FDH in 50 MM CHES (pH 9.5) with 150 mM NaCl.	6.42Å<br><br>0.247, 0.302<br><br>0.52 (at 6.17Å)<br>14.3 (2.1)<br><br>0.998 (0.827)	I432	154.10				Bailey & Tezcan (2020)
7K4Y	291K	343K	30% (w/v) PEG 6,000, 100 mM PIPES, pH<br>7.0, and 10 mM DTT.	1.80Å<br><br>0.1619, 0.2005<br><br>0.61 (at 1.81Å)<br>7.38 (0.99)<br><br>0.987 (0.286)	$P2_12_12_1$	49.71<br>68.12 73.72	7 bound waters removed by PDB REDO out of 78.		Authors provide additional notes in SI of refinement.	Otten et al. (2020)
8D8F	295K	325K	1.2 M NaCl and 100 mM ACONA pH 4.6,	1.49Å<br>0.145, 0.202<br><br>1.07 (at 1.50Å)<br>5.290 (0.020)<br><br>0.788	$P4_32_12$	79.61 37.72	2 bound waters removed by PDB REDO out of 29.			de Sá Ribeiro & Lima (2023)
8D8G	295K	325K	1.2 M NaCl and 100 mM	1.49Å	$P4_32_12$	79.81	1 bound			de Sá

			ACONA pH 4.6,	0.143, 0.199<br><br>1.08 (at 1.50Å)<br>5.330 (0.030)<br><br>0.535		37.71	water removed by PDB REDO out of 30.			Ribeiro & Lima (2023)
8D8H	295K	325K	1.2 M NaCl and 100 mM ACONA pH 4.6.	1.50Å<br>0.187, 0.213<br><br>1.45 (at 1.50Å)<br>5.340 (0.050)<br><br>0.684	$P4_32_12$	79.89 37.80	No bound waters removed by PDB REDO out of 26.			de Sá Ribeiro & Lima (2023)
8SOG[&]	298K	313K	Proteinase K was dissolved at pH 7.5 to 30 mg/ml in a 50 mM TRIS buffer. The protein was crystallized by mixing equal amounts of protein solution with 1.2 M ammonium sulfate.	1.13Å<br><br>0.1189, 0.1196<br><br>1.16 (at 1.13Å)<br>11.17 (1.13)<br><br>0.999 (0.519)	$P4_32_12$	68.40 103.71	89 bound waters removed by PDB REDO out of 225.		Refinement parameters obtained from PDB file.	Du et al. (2023)
8SOU	298K	363K	Proteinase K was dissolved at pH 7.5 to 30 mg/ml in a 50 mM TRIS buffer . The protein was crystallized by mixing equal amounts of protein solution with ammonium sulfate.	1.54Å<br><br>0.1728, 0.1756<br><br>1.21 (at 1.55Å)<br>6.54 (0.79)<br><br>0.999 (0.383)	$P4_32_12$	68.46 104.97	28 bound waters removed by PDB REDO out of 92.			Du et al. (2023)
8SOV	298K	353K	Proteinase K was	1.29Å	$P4_32_12$	68.36 104.04	55 bound			Du et al.

			dissolved at pH 7.5 to 30 mg/ml in a 50 mM TRIS buffer. The protein was crystallized by mixing equal amounts of protein solution with 1.2 m ammonium sulfate.	0.1307, 0.1318<br>1.15 (at 1.29Å)<br>13.89 (0.87)<br>0.999 (0.333)			waters removed by PDB REDO out of 148.			(2023)
8SPL	298K	343K	Proteinase K was dissolved at pH 7.5 to 30 mg/ml in a 50 mM TRIS buffer. The protein was crystallized by mixing equal amounts of protein solution with 1.2 m ammonium sulfate.	1.21Å<br>0.1745, 0.1754<br>1.06 (at 1.21Å)<br>12.72 (065)<br>1 (0.339)	$P4_32_12$	68.40 104.11	22 bound waters removed by PDB REDO out of 126. Two bound waters could be reinstated and several others placed.			Du et al. (2023)
8SQV	298K	333K	Proteinase K was dissolved at pH 7.5 to 30 mg/ml in a 50 mM TRIS buffer. The protein was crystallized by mixing equal amounts of protein solution with 1.2 m ammonium sulfate.	1.22Å<br>0.1315, 0.1325<br>1.14 (at 1.22Å)<br>11.29 (0.76)<br>0.999 (0.305)	$P4_32_12$	68.07 103.28	37 bound waters removed by PDB REDO out of 158. Two could be reinstated.			Du et al. (2023)
9JD2	293.2K	353K	The mixture of protein solution and agarose was filled into a glass capillary, and the capillary was immersed in the	1.62Å<br>0.1228, 0.1676<br>1.31 (at 1.62Å)<br>9.8 (1.1)	H32	79.05 233.48		Several bound waters could be added (none were deleted by PDB REDO). Several peaks sit	Refinement parameters obtained from PDB file.	Lizuka et al. (2024)<br>*To be published.*

			reservoir solution for crystallization. The reservoir solution contained: 15%(v/v) PEG 200,0.1 M phosphate citrate pH 5.4, 5 mM MTA.	0.999 (0.344)				on the symmetry 3 and 2 fold axes.		
9JHV	293.2K	343K	The mixture of protein solution and agarose was filled into a glass capillary, and the capillary was immersed in the reservoir solution for crystallization. The reservoir solution contained: 15%(v/v) PEG 200,0.1 M phosphate citrate pH 5.4, 5 mM MTA.	1.65Å<br><br>0.1182, 0.1674<br><br>1.28 (at 1.65Å)<br>10 (1.1)<br><br>0.997 (0.339)	H32	78.85 233.37		Several bound waters could be added (none were deleted by PDB REDO).		Lizuka et al. (2024) *To be published.*
9G5X	295K	318K	(35% (w/v) PEG 3350, 200 mM $LiSO_4$ and 10 mM HEPES / NaOH, pH 7.5)	1.70Å<br><br>0.1453, 0.1824<br><br>2.54 (at 1.70Å).<br>8.57(2.88).<br>98.64(86.29).	I222	99.25 94.20 103.05	55 bound waters removed by PDB REDO out of 287. 22 bound waters of the depositors could be reinstated			Schulz et al. (2025)

							into the PDB REDO Fo-Fc map peaks.			
9G61	295K	323K	(35% (w/v) PEG 3350, 200 mM $LiSO_4$ and 10 mM HEPES / NaOH, pH 7.5)	1.70Å. 0.1590, 0.1903 1.28 (at 1.70Å). 4.87(1.14). 96.25(45.20).	I222	94.20 103.05 99.25	54 bound waters removed by PDB REDO out of 256. 13 bound waters of the depositors could be reinstated into the PDB REDO Fo-Fc map peaks.			Schulz et al. (2025)
9G6O	295K	318K	(35% (w/v) PEG 3350, 200 mM $LiSO_4$ and 10 mM HEPES / NaOH, pH 7.5)	1.70Å. 0.1479, 0.1859 2.41 (at 1.70Å). 6.39(2.67). 96.44(79.42).	I222	94.20 103.05 99.25	53 bound waters removed by PDB REDO out of 270. 13 bound waters of the depositors could be reinstated into the PDB REDO Fo-Fc map peaks.			Schulz et al. (2025)
9G6P	295K	323K	(35% (w/v) PEG 3350, 200 mM $LiSO_4$ and 10 mM HEPES / NaOH, pH 7.5)	1.70Å. 0.1744, 0.2153 1.18 (at 1.70Å).	I222	94.20 103.05 99.25	43 bound waters removed by PDB REDO out of 258.			Schulz et al. (2025)

				3.24(0.93).<br>89.5(36.2).			4 bound waters of the depositors could be reinstated into the PDB REDO Fo-Fc map peaks.			
9G7Z	293K	323K	CTX-M-14 solution (22 mg/ml) was mixed with 45% precipitant solution (40% PEG8000, 200mM lithium sulfate, 100mM sodium acetate, pH 4.5) and with 5% undiluted seed stock in batch crystallization setups	1.70Å<br><br>0.160, 0.186.<br><br>1.23 (at 1.70Å).<br>5.65(1.04).<br>0.967(0.481).	$P3_221$	41.85 232.85	45 bound waters removed by PDB REDO out of 134. 4 bound waters of the depositors could be reinstated into the PDB REDO Fo-Fc map peaks.			Schulz et al. (2025)
9I7L	295K	323K	(35% (w/v) PEG 3350, 200 mM $LiSO_4$ and 10 mM HEPES/ NaOH, pH 7.5)	1.70Å.<br><br>0.1690, 0.2066<br><br>1.16 (at 1.70Å).<br>3.38(0.87).<br>91.40(33.03).	I222	94.20 103.05 99.25	46 bound waters removed by PDB REDO out of 252. 4 bound waters of the depositors could be reinstated into the PDB REDO Fo-Fc			Schulz et al. (2025)

							map peaks.			
9JKU	293.2K	333K	Counter diffusion crystallization method using a glass capillary immersed in the reservoir solution for crystallization. 15%(v/v) PEG#200, 0.1 M phosphate citrate pH 5.4, 5 mM MTA	1.61Å<br><br>0.1398, 0.1683<br><br>23.7(2.6)<br>N/A<br>0.999(0.672).	H32	78.924<br>78.924<br>233.512				(Lizuka *et al.*, 2025*c*) *To be published.*
9JRZ	293.2K	323K	Counter diffusion crystallization method. 15%(v/v) PEG#200, 0.1 M phosphate citrate pH 5.4, 5 mM MTA	1.69Å<br><br>0.1097, 0.1439<br><br>18.9(1.9)<br>N/A<br>0.999(0.680).	H32	78.927<br>78.927<br>233.574				(Lizuka *et al.*, 2025*b*) *To be published.*
9JTG	293.2K	353K	Counter diffusion crystallization method. 15%(v/v), PEG#200, 0.1 M phosphate citrate pH 5.4	1.68Å<br><br>0.1400, 0.1687<br><br>21.6(2.7)<br>N/A<br>0.999(0.692).	H32	79.127<br>79.127<br>233.26				(Lizuka *et al.*, 2025*a*) *To be published.*

Footnotes: [&] Although 313K it is part of a series of structures i.e. including 8SOU and 8SOV.

Note added to manuscript proof: Doukov et al (2025) report observations on rubredoxin crystal structures studied up to 120°C (PDB code: 9Y2Y for 393K, structure details unreleased at time of this manuscript's acceptance).

Table S3 . CryoEM study at 37°C (flash frozen).

**PDB code**	**CryoEM study core details**	**Title of PDB entry**		**PDB Validation Report**	**Ref.**
9B8W	3.10Å resolution.<br>4 chains.<br>8 Ca ions.	Cryo-EM structure of the human TRPM4 in complex with calcium at 37°C		Each of the 8 Ca ions are 5 coordinate in the model.	Hu et al. (2024)
9B92	3.50Å resolution.<br>4 chains.<br>4 Ca ions.	Cryo-EM structure of the human TRPM4 in complex with calcium at 18°C			Hu et al. (2024)

Table S4 Crystallographic parameters of select small molecules found within the CSD drug subset.

	Temp. (K)	Cell parameters Å a	b	c	° α	β	γ	Vol.	Space group	Solvent	Additional molecule	Disordered	Interaction comment
R-Methionine													
ZOTMOA	100	16.4457(10)	4.6941(3)	29.1646(16)	90	104.877(3)	90	2175.97	$P2_1$	None	S-ethylcysteine	None	H-bonds found in the amino-acid portion of the molecule
DLMETA02	333	9.89(2)	4.70(2)	16.74(3)	90	102.3(7)	90	760.264	$P2_1/a$	None	None	None	H-bonds found in the amino-acid portion of the molecule
URODIP06	355	63.73(3)	4.799(2)	9.857(5)	90	91.113(5)	90	3014.1	C2	None	L-norvaline	Both	H-bonds found in the amino-acid portion of the molecule
URODIP05	330	9.849(4)	4.776(2)	16.236(7)	90	103.123(5)	90	743.778	$P2_1$	None	L-norvaline	Both	H-bonds found in the amino-acid portion of the molecule
URODIP07	380	32.44(2)	4.815(3)	9.882(7)	90	99.242(7)	90	1523.52	C2	None	S-2-aminopentanoic acid	Both	H-bonds found in the amino-acid portion of the molecule
VUQNAY02	379	9.886(12)	4.817(6)	16.59(2)	90	96.485(15)	90	784.975	$P2_1$	None	L-norleucine	Both	H-bonds found in the amino-acid portion of the molecule
VUQNAY03	387	9.8546(13)	4.8053(6)	33.303(5)	90	97.977(4)	90	1561.78	$P2_1$	None	L-norleucine	Both	H-bonds found in the amino-acid portion of the molecule
VUQNAY04	393	9.922(11)	4.842(6)	17.01(2)	90	101.132(14)	90	801.824	$P2_1$	None	L-norleucine	Both	H-bonds found in the amino-acid portion of the molecule
Glycine													
GLYCIN95	100	5.08760(10)	11.8092(2)	5.46150(10)	90	111.9920(10)	90	304.254	$P2_1/n$	None	None	None	Interactions across the whole molecule
GLYCIN22	313	5.1012(3)	11.9651(9)	5.4604(4)	90	111.763(5)	90	309.528	$P2_1/n$	None	None	None	Interactions across the whole molecule
GLYCIN23	323	5.1026(3)	11.9752(9)	5.4602(4)	90	111.757(5)	90	309.876	$P2_1/n$	None	None	None	Interactions across the whole molecule
GLYCIN24	427	5.1074(3)	12.0775(9)	5.4596(4)	90	111.827(5)	90	312.63	$P2_1/n$	None	None	None	Interactions across the whole molecule
L-pyroglutamic acid													
PYRGLU11	100	7.8818(14)	8.7859(15)	8.9967(16)	90	115.426(2)	90	562.665	$P2_1/c$	None	None	None	Interactions across the whole molecule
LPYGLU01	331	9.0379(9)	13.4729(14)	14.6797(13)	90	90	90	1787.5	$P2_12_12_1$	None	None	None	Interactions across the whole molecule
LPYGLU07	330	9.0390(10)	13.4680(10)	14.6700(10)	90	90	90	1785.89	$P2_12_12_1$	None	None	None	Interactions across the whole molecule
LPYGLU08	353	4.4135(7)	9.1662(14)	14.753(2)	90	90	90	596.833	$P2_12_12_1$	None	None	None	Interactions across the whole molecule
VUPROR	354	9.1584(14)	4.3947(4)	14.732(2)	90	90	90	592.94	$P2_12_12_1$	None	None	None	Interactions across the whole molecule
L-asparagine													
ASPARM05	100	5.584(1)	9.735(1)	11.701(2)	90	90	90	636.069	$P2_12_12_1$	One Water	None	None	Interactions across the whole molecule
ASPARM22	313	5.5811(2)	9.8272(5)	11.8045(5)	90	90	90	647.437	$P2_12_12_1$	One Water	None	None	Interactions across the whole molecule
ASPARM23	333	5.5789(2)	9.8426(4)	11.8127(5)	90	90	90	648.646	$P2_12_12_1$	One Water	None	None	Interactions across the whole molecule
ASPARM24	353	5.5736(2)	9.8650(5)	11.8179(6)	90	90	90	649.79	$P2_12_12_1$	One Water	None	None	Interactions across the whole molecule
ASPARM25	373	5.5746(3)	9.8784(6)	11.8396(7)	90	90	90	651.985	$P2_12_12_1$	One Water	None	None	Interactions across the whole molecule

References

Bailey, J. B. & Tezcan, F. A. (2020). *J Am Chem Soc* 142, 17265–17270.

Chevrier, B., Dock, A. C., Hartmann, B., Leng, M., Moras, D., Thuong, M. T. & Westhof, E. (1986). *J Mol Biol* 188, 707–719.

Deck, S. L. , X. M. , A. C. , M. S. K. *To Be Published*. Temperature Gradient Structures of Oncogenic KRAS G12C Mutants.

Doukov, T., Herschlag, D. & Yabukarski, F. (2023). *Acta Cryst. Section D Structural Biology* 79, 212–223.

Doukov, T., Leontyev, I., Jenney Jr, F.E., George, D. and Cramer, S.P., (2025). Angewandte Chemie International Edition, p.e20302. doi.org/10.1002/anie.202520302

Du, S., Wankowicz, S. A., Yabukarski, F., Doukov, T., Herschlag, D. & Fraser, J. S. (2023). Vol. *688*, *Methods in Enzymology*. pp. 223–254. Academic Press.

Ebrahim, A., Riley, B. T., Kumaran, D., Andi, B., Fuchs, M. R., McSweeney, S. & Keedy, D. A. (2022). *IUCrJ* 9, 682–694.

Fukuda, Y. & Inoue, T. (2015). *Chemical Communications* 51, 6532–6535.

Greisman, J. B., Dalton, K. M., Brookner, D. E., Klureza, M. A., Sheehan, C. J., Kim, I.-S., Henning, R. W., Russi, S. & Hekstra, D. R. (2024). *Proc. Natl. Acad. Sci.* 121, https://doi.org/10.1073/pnas.2313192121.

Guerrero, L., Ebrahim, A., Riley, B. T., Kim, M., Huang, Q., Finke, A. D. & Keedy, D. A. (2023). *BioRxiv* 1–27.

Hu, J., Park, S. J., Walter, T., Orozco, I. J., O‘Dea, G., Ye, X., Du, J. & Lü, W. (2024). *Nature* 630, 509–515.

Jacobs, F. J. F., Helliwell, J. R. & Brink, A. (2024). *Chemical Communications* 60, 14030–14033.

Keedy, D. A., Kenner, L. R., Warkentin, M., Woldeyes, R. A., Hopkins, J. B., Thompson, M. C., Brewster, A. S., Van Benschoten, A. H., Baxter, E. L., Uervirojnangkoorn, M., McPhillips, S. E., Song, J., Alonso-Mori, R., Holton, J. M., Weis, W. I., Brunger, A. T., Soltis, S. M., Lemke, H., Gonzalez, A., Sauter, N. K., Cohen, A. E., van den Bedem, H., Thorne, R. E. & Fraser, J. S. (2015). *Elife* 4, 1–6.

Lizuka, Y., Kikuchi, M., Yamauchi, T. & Tsunoda, M. (2024). *To Be Published.* https://doi.org/10.2210/pdb9jd2/pdb.

Lizuka, Y., Kikuchi, M., Yamauchi, T. & Tsunoda, M. (2025*a*). *Worldwide Protein Data Bank* https://doi.org/10.2210/pdb9jtg/pdb.

Lizuka, Y., Kikuchi, M., Yamauchi, T. & Tsunoda, M. (2025*b*). *Worldwide Protein Data Bank* https://doi.org/10.2210/pdb9jrz/pdb.

Lizuka, Y., Kikuchi, M., Yamauchi, T. & Tsunoda, M. (2025*c*). *Worldwide Protein Data Bank* https://doi.org/10.2210/pdb9jku/pdb.

McLeod, M. J., Barwell, S. A. E., Holyoak, T. & Thorne, R. E. (2025). *Structure* 33, 924-934.e2.

Oki, H., Matsuura, Y., Komatsu, H. & Chernov, A. A. (1999). *Acta Cryst. D: Biological Crystallography* 55, 114–121.

Otten, R., Pádua, R. A. P., Bunzel, H. A., Nguyen, V., Pitsawong, W., Patterson, M., Sui, S., Perry, S. L., Cohen, A. E., Hilvert, D. & Kern, D. (2020). *Science (1979)* 370, 1442–1446.

Paolillo, M., Ferraro, G., Gumerova, N. I., Pisanu, F., Garribba, E., Rompel, A. & Merlino, A. (2025). *Inorg Chem Front* 1–16.

Parkins, A., Pilien, A. V. R., Wolff, A. M., Argueta, C., Vargas, J., Sadeghi, S., Franz, A. H., Thompson, M. C. & Pantouris, G. (2024). *J Med Chem* 67, 7359–7372.

de Sá Ribeiro, F. & Lima, L. M. T. R. (2023). *Biophys. Chem.* 298, 1–12.

Sammons, R. M., Perry, N. A., Li, Y., Cho, E. J., Piserchio, A., Zamora-Olivares, D. P., Ghose, R., Kaoud, T. S., Debevec, G., Bartholomeusz, C., Gurevich, V. V, Iverson, T. M., Giulianotti, M., Houghten, R. A. & Dalby, K. N. (2019). *ACS Chem Biol* 14, 1183–1194.

Schuller, M., Correy, G. J., Gahbauer, S., Fearon, D., Wu, T., Díaz, R. E., Young, I. D., Carvalho Martins, L., Smith, D. H., Schulze-Gahmen, U., Owens, T. W., Deshpande, I., Merz, G. E., Thwin, A. C., Biel, J. T., Peters, J. K., Moritz, M., Herrera, N., Kratochvil, H. T., Aimon, A., Bennett, J. M., Brandao Neto, J., Cohen, A. E., Dias, A., Douangamath, A., Dunnett, L., Fedorov, O., Ferla, M. P., Fuchs, M. R., Gorrie-Stone, T. J., Holton, J. M., Johnson, M. G., Krojer, T., Meigs, G., Powell, A. J., Rack, J. G. M., Rangel, V. L., Russi, S., Skyner, R. E., Smith, C. A., Soares, A. S., Wierman, J. L., Zhu, K., O'Brien, P., Jura, N., Ashworth, A., Irwin, J. J., Thompson, M. C., Gestwicki, J. E., von Delft, F., Shoichet, B. K., Fraser, J. S. & Ahel, I. (2021). *Sci Adv* 7, 1–23.

Schulz, E. C., Prester, A., von Stetten, D., Gore, G., Hatton, C. E., Bartels, K., Leimkohl, J.-P., Schikora, H., Ginn, H. M., Tellkamp, F. & Mehrabi, P. (2025). *Nat Commun* 16, 1–12.

Tito, G., Ferraro, G., Garribba, E. & Merlino, A. (2025a). *Chemistry – A European Journal* 31, 1.

Tito, G., Ferraro, G. & Merlino, A. (2025b). *Int J Mol Sci* 26, 1–11.
